# Influence of SCA on clinical outcomes and cervical alignment after laminoplasty in patients with multilevel cervical spondylotic myelopathy

**DOI:** 10.1186/s13018-021-02200-3

**Published:** 2021-01-12

**Authors:** Zheng Wang, Zhi-Wei Wang, Xi-Wen Fan, Zhen Liu, Jia-Yuan Sun, Wen-Yuan Ding, Da-Long Yang

**Affiliations:** Department of Spinal Surgery, The Third Hospital of Hebei Medical University, 139 Ziqiang Road, Shijiazhuang, 050051 People’s Republic of China

**Keywords:** Spino-cranial angle, Laminoplasty, Multilevel cervical spondylotic myelopathy, Neck Disability Index, Sagittal alignment

## Abstract

**Background:**

To study the impact of changes in spino-cranial angle (SCA) on sagittal alignment and to investigate the relationship between SCA and Neck Disability Index (NDI) scores after laminoplasty (LP)

**Material and methods:**

In total, 72 patients with multilevel cervical spondylotic myelopathy (MCSM) after laminoplasty (LP) were retrospectively enrolled. Based on the optimal cut-off values of preoperative SCA, patients were classified into low SCA and high SCA groups. Radiographic data were measured, including spino-cranial angle (SCA), T1-slope (T1s), C2–7 lordosis (CA), T1s minus CA (T1sCA), and C2–7 sagittal vertical axis (cSVA). JOA and NDI scores were both applied to assess postoperative and follow-up clinical efficacy. Pearson correlation coefficient and linear regression analysis were respectively calculated between radiographic data and between SCA and NDI.

**Results:**

The preoperative SCA was significantly correlated with T1s (*r* = − 0.795), CA (*r* = − 0.857), and cSVA (*r* = 0.915). A receiver operating characteristic (ROC) curve model predicted a threshold of SCA (value of 85.2°). At the follow-up period, patients with lower SCA had a higher T1s and CA and a lower cSVA, simultaneously accompanied by greater △T1s, △CA, and △cSVA. The linear regression model demonstrated that SCA in the higher group was positively correlated with NDI, and patients with higher SCA had worse NDI scores (pre: *p* < 0.001; post: *p* < 0.001; F/U: *p* = 0.003) and greater changes of NDI (post: *p* < 0.010; F/U: *p* = 0.002).

**Conclusion:**

SCA may be a good predictor of evaluating sagittal balance and planning surgery. Changes in sagittal alignment in the low SCA group were affected more easily, and a higher SCA was associated with worse quality of life. Laminoplasty could be a good choice for patients with lower SCA.

## Introduction

Laminoplasty (LP), a commonly used indirect posterior decompression surgery for treating multilevel cervical spondylotic myelopathy (MCSM) degenerative diseases, is often related to clinical outcomes [1, 2]. It achieves sufficient decompression by drifting the spinal cord backwards [3]. Analysis of cervical alignment parameters is important to evaluate sagittal balance and to predict clinical outcomes. Recently, three pivotal sagittal parameters, spino-cranial angle (SCA), T1-slope (T1s), and C2–7 sagittal vertical axis (cSVA), were reported to be the focal point of future research [4]. Among them, SCA is a neglected but essential parameter, defined as the angle between a line from the sella turcica centre and C7 endplate and the C7 plateau line. Studies also reported that SCA is significantly correlated with many sagittal parameters [5]. Additionally, some cervical sagittal parameters are relevant to the health-related quality of life based on previously published literature [6, 7]. However, whether SCA can be used as a predictor to affect the cervical alignment and clinical outcomes of patients with multilevel cervical spondylotic myelopathy (MCSM) following laminoplasty is unclear. Therefore, in the present study, we classified SCA into two categories based on the cut-off value to determine cervical sagittal balance and analyze the relationship between SCA and clinical outcomes after laminoplasty.

## Methods

### Patient population

A retrospective study of radiographic and clinical outcomes was performed in patients with multilevel cervical spondylotic myelopathy who received laminoplasty in the Department of Spinal Surgery, the Third Hospital of Hebei Medical University, from January 2011 to December 2016. The inclusion criteria were as follows: (1) three or more levels of compression were observed by MRI examination; (2) integrated radiographic and clinical data were collected; and (3) follow-up period of more than 24 months. The exclusion criteria were as follows: (1) previous cervical surgery; (2) cervical spine fracture, infection, and tumour; (3) ankylosing spondylitis and rheumatoid arthritis; (4) follow-up period less than 2 years; and (5) radiological parameters were too unclear to measure. Health-related outcomes, including Japanese Orthopaedic Association (JOA) (score 0–17) and Neck Disability Index (NDI) (range 0–50), were computed preoperatively, postoperatively, and at the follow-up period.

### Radiographic measurements

**Fig. 1:**
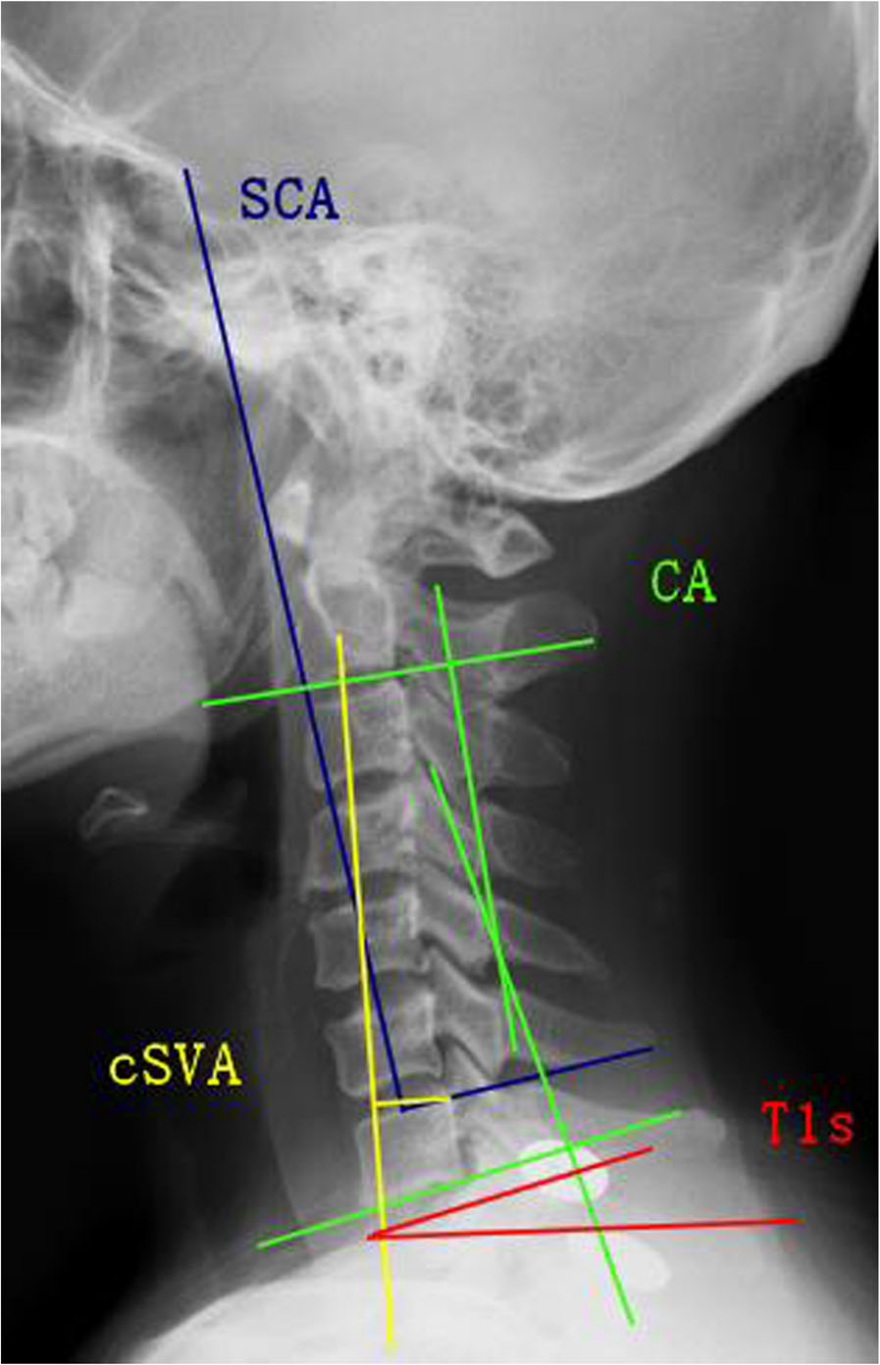
Spino-cranial angle (SCA), the angle is defined between the C7 slope and the straight line joining the middle of the C7 end plate and the middle of the sella turcica. T1-slope (T1s), angle between a horizontal line and the superior endplate of T1 or C7. C2-C7 lordosis (CA), angle between the lower plate of C2 and the lower plate of C7. C2-C7 SVA (cSVA), the distance from the posterior, superior corner of C7 to the plumbline from the centroid of C2

Imaging results were calculated from standard lateral X-rays. All concerning sagittal parameters were evaluated (Fig. 1): (1) spino-cranial angle (SCA), the angle between the C7 slope and the straight line joining the midpoint of the C7 endplate and the midpoint of the sella turcica; (2) T1-slope (T1s), the angle between a horizontal line and the upper endplate of T1; (3) C2-7 lordosis (CA), the angle defined from lower endplate of C2 to lower endplate of C7; (4) C2-7 sagittal vertical axis (cSVA), the distance between the C2 plumb line and the posterior upper endplate of C7; (5) T1s minus CA (T1sCA), the angle of T1-slope minus C2–C7 lordosis; (6) △, the difference values between the preoperative and postoperative or preoperative and follow-up visit for each parameter, such as SCA, T1s, CA, cSVA, and T1sCA.

### Statistical analysis

**Fig. 2:**
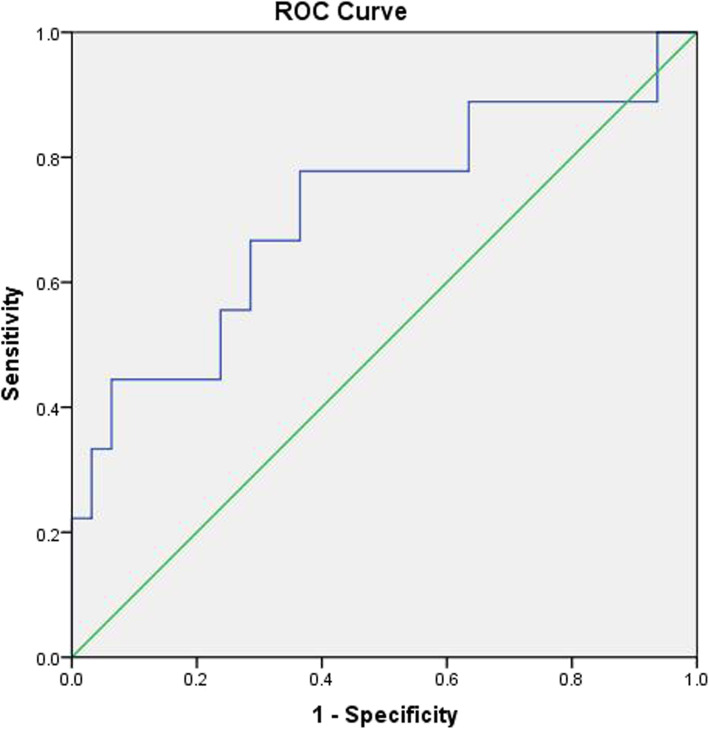
ROC curves of cut-off values of SCA for predicting sagittal balance. AUC result for cut-off values of SCA was 0.716. AUC, area under the curve; ROC, receiver operating characteristics; SCA, spino-cranial angle

Receiver operating characteristic (ROC) curve was used to analyze the capacity of preoperative SCA to predict cervical sagittal alignment (Fig. 2), and patients were then divided into two categories based on the selected optimal cut-off value of preoperative SCA. All data were statistically calculated by the SPSS software (version 22.0; SPSS Inc., Chicago, IL, USA) and presented as the means ± standard deviation. Pearson correlation coefficient was compared between SCA and other radiographic sagittal parameters. Linear regression analysis was applied to assess the relationships between SCA and NDI scores. Independent samples *t* test or Mann-Whitney *U* test was used to compare cervical sagittal alignment parameters and health-related outcomes between two groups. *P* < 0.05 was considered statistically significant.

## Results

### Analysis of ROC curve and cut-off value

As a criterion for judging balance, T1sCA was applied to assess sagittal balance (T1sCA ≤ 20°, cervical balance; > 20°, cervical imbalance). Analysis of the ROC curve for sagittal balance revealed that preoperative SCA of 85.2° was considered the optimal cut-off value. The AUC and *P* value were 0.716 and 0.051, respectively, for the cut-off value of SCA (Fig. 2).

### Comparison of patient characteristics based on preoperative SCA

**Fig. 3:**
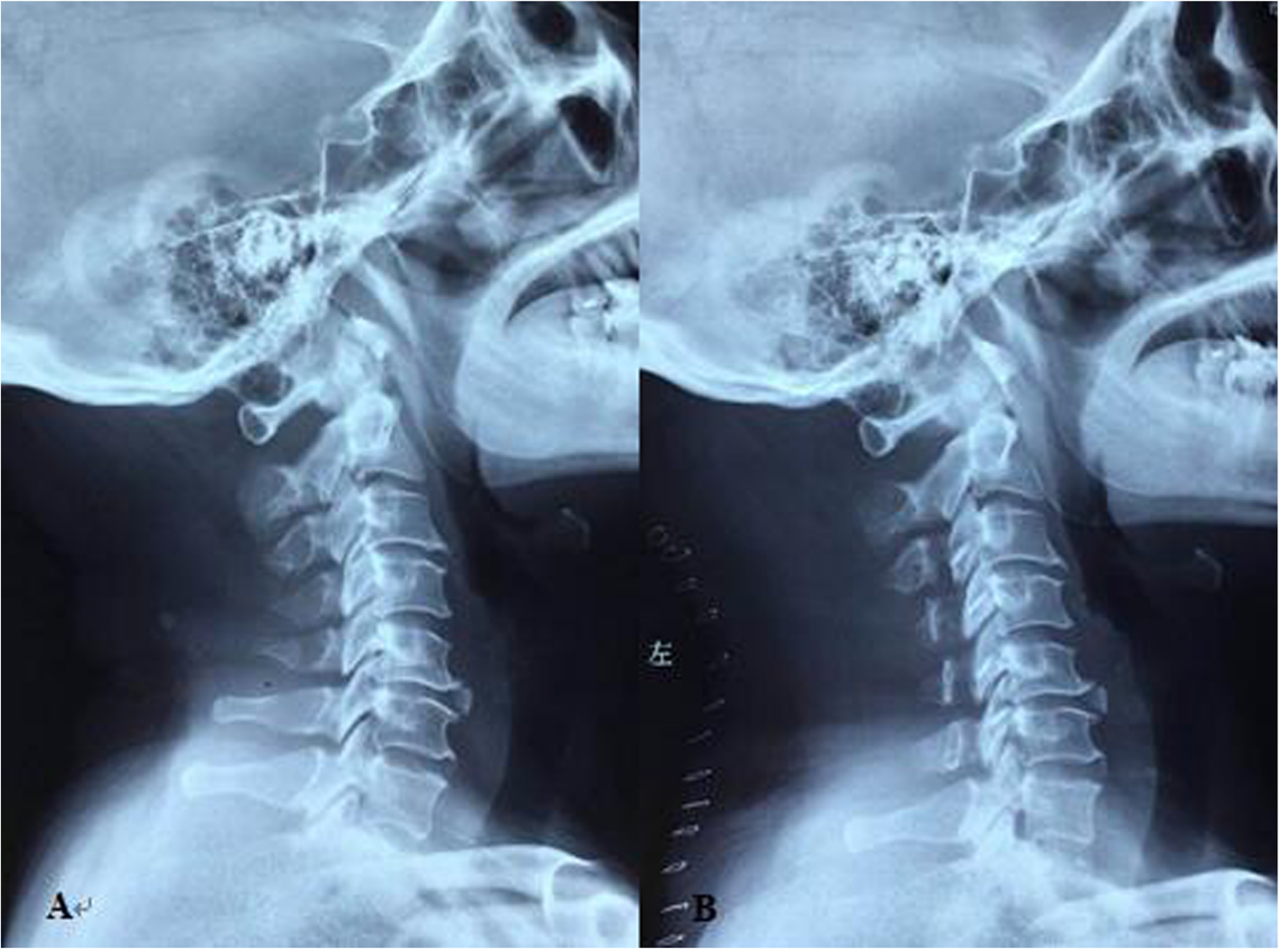
Laminoplasty was performed to release the compression. Lateral X-ray of cervical spine was taken in a 50-year-old male patient with MCSM preoperatively **a** and at the 2-year follow-up visit **b**, and sagittal parameters were corrected appropriately

Seventy-two patients were identified in this study. Their demographic data are summarized in Table 1. All the surgeries were completed successfully (Fig. 3).The average length of hospitalization (LOH) was 7.54 ± 1.50 in the low SCA group and 8.90 ± 1.58 in the high SCA group, which was statistically significant (*P* = 0.002). Other variables, such as age, sex, operation time, operative segments, and blood loss, were not statistically significant between the two groups.

**Table 1:** Patient characteristics

	LG (*n* = 41)	HG (*n* = 31)	*P* value
No. of patients	41	31	
Age (year)	59.34 ± 7.95	57.58 ± 9.92	0.406
Sex (male/female)	22/19	15/16	0.658
Operation time (min)	125.85 ± 30.58	121.61 ± 27.58	0.766
Length of hospitalization	7.54 ± 1.50	8.90 ± 1.58	0.002
Operative segments	3.29 ± 0.60	3.45 ± 0.68	0.229
Blood loss	182.68 ± 57.58	195.48 ± 64.54	0.459

*LG* low SCA group, *HG* high SCA group, *SCA* spino-cranial angle

### Correlation between SCA and other sagittal parameters

Significant correlations were found between SCA and T1-slope (T1s) (*r* = − 0.795, *P* < 0.001), between SCA and C2-C7 lordosis (CA) (*r* = − 0.857, *P* < 0.001), and between SCA and C2-C7 SVA (cSVA) (*r* = 0.915, *P* < 0.001). No correlation was shown between SCA and T1sCA (*r* = 0.072, *P* = 0.546). *P* values and correlations between the different parameters are shown in Table 2.

**Table 2:** Correlation between pre-SCA and other sagittal parameters

Parameters	Correlation	*P* value	Significance
T1s (°)	− 0.795	< 0.001	S
CA (°)	− 0.857	< 0.001	S
cSVA (mm)	0.915	< 0.001	S
T1sCA (°)	0.072	0.546	NS

*SCA* spino cranial angle, *T1s* T1-Slope, *CA* C2–7 lordosis angle, *cSVA* C2–7 sagittal vertical axis, *T1sCA* T1s minus CA

### Correlation between SCA and health-related quality of life (HRQOL) scores

According to linear regression model analysis, SCA was not significantly correlated with the JOA scores. However, for NDI, the results were rather interesting. Whether at the preoperative, postoperative, or follow-up period, SCA in the higher group was all positively correlated with the NDI scores (pre: *r* = 0.876, *P* < 0.001; post: *r* = 0.414, *P* = 0.020; F/U: *r* = 0.431, *P* = 0.015). However, in the lower SCA group, there was no significant correlation between SCA and NDI scores preoperatively (*r* = 0.159, *P* = 0.320), postoperatively (*r* = 0.055, *P* = 0.731), or at the follow-up period (*r* = − 0.005, *P* = 0.947) (Table 3).

**Table 3:** Correlation between SCA and NDI scores in the two groups

		LG	HG
Pre	*r*	0.159	0.876
	*P*	0.320	< 0.001
Post	*r*	0.055	0.414
	*P*	0.731	0.020
F/U	*r*	− 0.005	0.431
	*P*	0.974	0.015

*Pre* preoperative, *Post* postoperative, *F/U* follow-up, *LG* low SCA group, *HG* high SCA group, *SCA* spino-cranial angle

### Comparison of clinical outcomes based on preoperative SCA

**Fig. 4:**
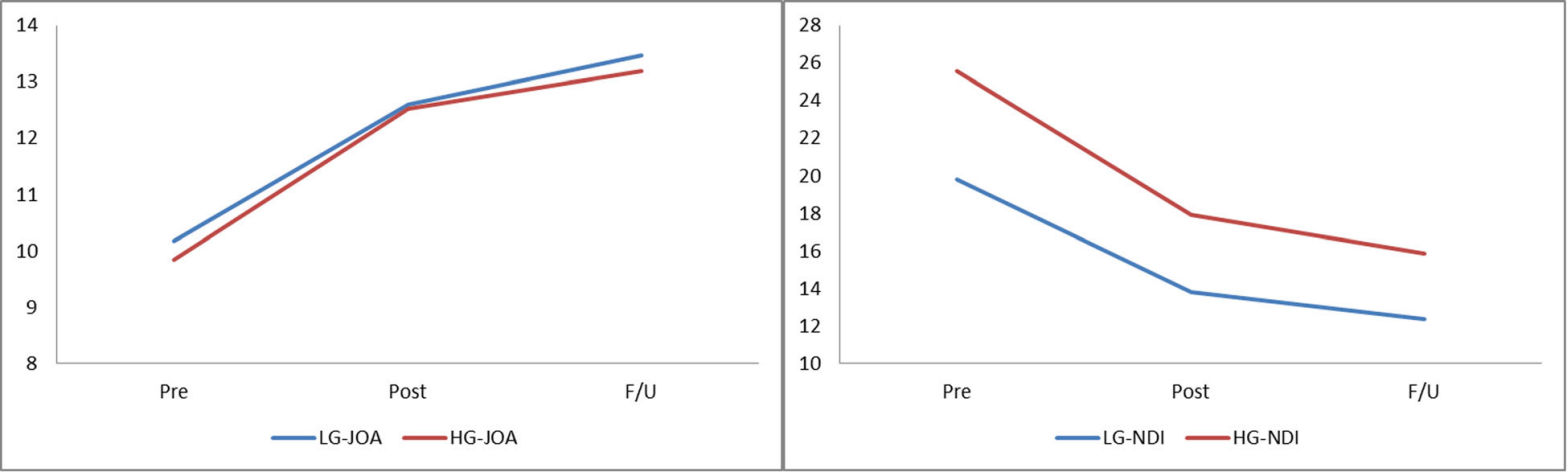
The change in the clinical results

Both groups showed significant improvement in JOA and NDI scores after laminoplasty. Compared with patients with lower SCA, patients in the higher SCA group expressed higher NDI at various time points (pre 25.58 ± 5.74 vs 19.80 ± 4.77, *P* < 0.001; post 17.90 ± 4.36 vs 13.78 ± 3.70, *P* < 0.001; F/U 15.84 ± 4.85 vs 12.39 ± 3.58, *P* = 0.003). The changes of NDI in the high SCA group exceeded those in the low SCA group (pre vs post: *P* = 0.010; pre vs F/U: *P* = 0.002). However, JOA scores and △JOA failed to reach significance between the two groups (Table 4). The changes in the above clinical results are shown in the form of a line chart (Fig. 4).

**Table 4:** Quality of clinical parameters

	LG (*n* = 41)	HG (*n* = 31)	*P* value
JOA			
Pre	10.17 ± 2.12	9.84 ± 2.18	0.589
Post	12.59 ± 1.34	12.52 ± 1.65	0.931
F/U 2 years	13.46 ± 0.81	13.19 ± 1.17	0.208
△JOA (pre vs post)	2.41 ± 1.58	2.68 ± 1.58	0.475
△JOA (pre vs F/U)	3.29 ± 2.15	3.35 ± 1.80	0.949
Pre vs F/U	< 0.001	< 0.001	
NDI			
Pre	19.80 ± 4.77	25.58 ± 5.74	< 0.001
Post	13.78 ± 3.70	17.90 ± 4.36	< 0.001
F/U 2 years	12.39 ± 3.58	15.84 ± 4.85	0.003
△NDI (pre vs post)	− 6.02 ± 2.29	− 7.68 ± 2.98	0.010
△NDI (pre vs F/U)	− 7.41 ± 2.93	− 9.74 ± 3.63	0.002
Pre vs F/U	< 0.001	< 0.001	

*LG* low SCA group, *HG* high SCA group, *JOA* Japanese Orthopaedic Association, *NDI* Neck Disability Index

### Comparison of radiographic parameters based on preoperative SCA

**Fig. 5:**
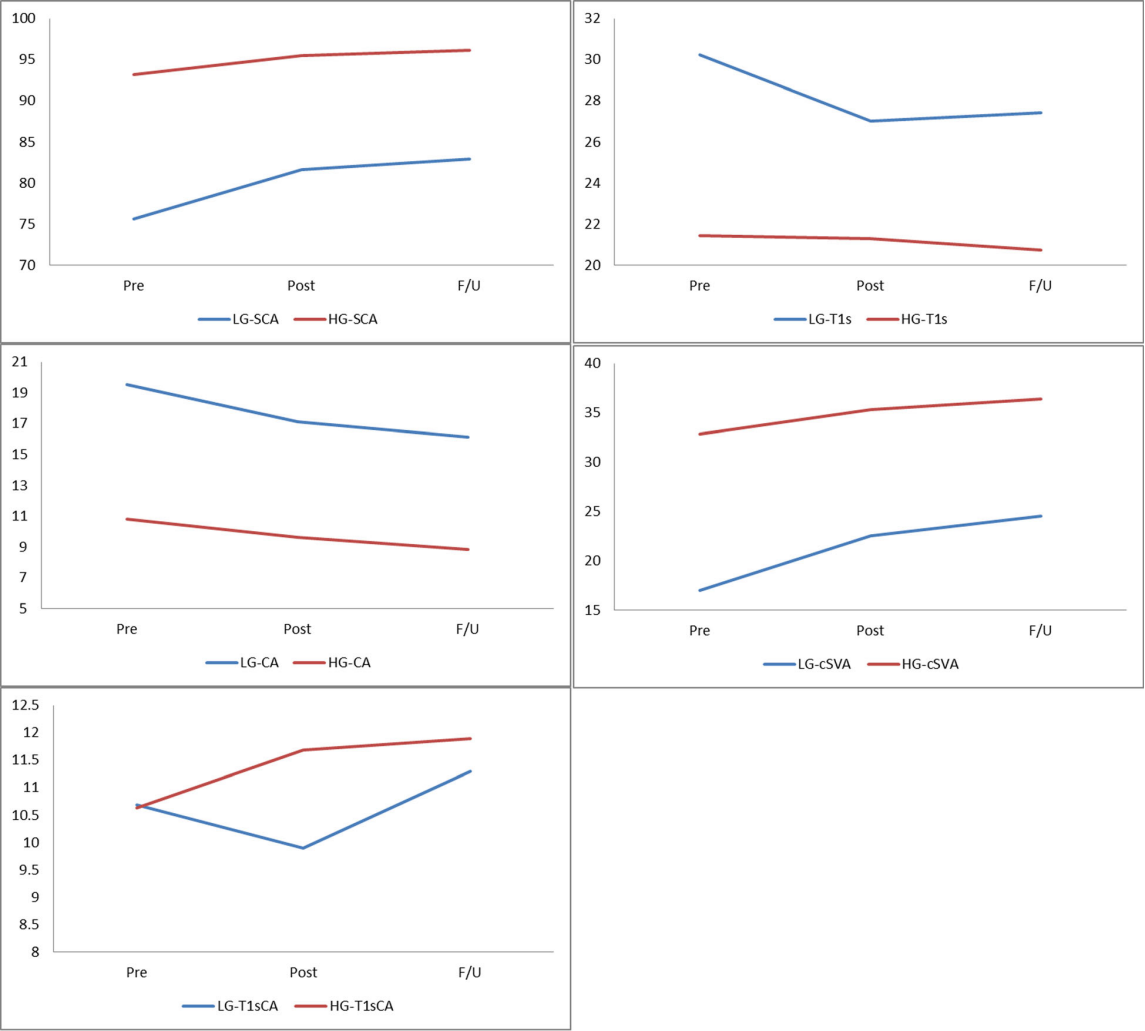
The change in the radiographic parameters

Tables 5 and 6 summarize the preoperative, postoperative, and follow-up values and changes of radiographic parameters. The mean ± standard deviation values of pre-SCA, post-SCA, and F/U-SCA were 75.65° ± 5.08°, 81.59° ± 7.76°, and 82.88° ± 7.42° in the low SCA group and 93.15° ± 5.25°, 95.45° ± 6.09°, and 96.17° ± 5.26° in the high SCA group, respectively, which all displayed significant differences (*P* < 0.001). Before laminoplasty, compared with patients in the low SCA group, patients with higher SCA had smaller T1s (*P* < 0.001) and CA (*P* < 0.001) and larger cSVA (*P* < 0.001). The trends were maintained at the postoperative (T1s: *P* < 0.001; CA: *P* < 0.001; cSVA: *P* < 0.001) and follow-up periods (T1s: *P* < 0.001; CA: *P* < 0.001; cSVA: *P* < 0.001). Unfortunately, preoperative T1sCA did not differ significantly between the two groups (*P* = 0.348), nor did the postoperative (*P* = 0.213) or follow-up T1sCA (*P* = 0.661) (Table 5). Interestingly, patients with lower preoperative SCA following laminoplasty had significantly greater alignment changes than those in the higher group at the postoperative and follow-up periods, such as △SCA (pre vs post: *P* = 0.019; pre vs F/U: *P* = 0.005), △T1s (pre vs post: *P* = 0.007; pre vs F/U: *P* = 0.028), and △CA (pre vs post: *P* = 0.005; pre vs F/U: *P* = 0.017). Concerning cSVA, although △cSVA (pre vs post) did not show the same significant trend as mentioned above (5.57 mm ± 5.84 mm vs 2.52 mm ± 6.89 mm) (*P* = 0.055), a significant difference was revealed between the two groups at the follow-up period (7.51 mm ± 8.03 mm vs 3.61 mm ± 5.76 mm) (*P* = 0.019). Unfortunately, a statistically significant difference in △T1sCA was not found between the two groups either after surgery or during the follow-up (Table 6). The changes in the above radiographic parameters are shown in the form of a line chart (Fig. 5).

**Table 5:** Comparison of cervical radiologic parameters

	LG (*n* = 41)	HG (*n* = 31)	*P* value
SCA (°)			
Pre	75.65 ± 5.08	93.15 ± 5.25	< 0.001
Post	81.59 ± 7.76	95.45 ± 6.09	< 0.001
F/U	82.88 ± 7.42	96.17 ± 5.26	< 0.001
T1s (°)			
Pre	30.23 ± 5.80	21.42 ± 4.28	< 0.001
Post	27.02 ± 5.52	21.28 ± 5.82	< 0.001
F/U	27.41 ± 4.51	20.73 ± 4.91	< 0.001
CA (°)			
Pre	19.55 ± 4.97	10.79 ± 5.45	< 0.001
Post	17.12 ± 4.75	9.60 ± 4.45	< 0.001
F/U	16.11 ± 5.07	8.84 ± 4.50	< 0.001
cSVA (mm)			
Pre	16.98 ± 5.52	32.81 ± 5.24	< 0.001
Post	22.55 ± 6.25	35.32 ± 7.49	< 0.001
F/U 2 years	24.49 ± 8.21	36.42 ± 6.02	< 0.001
T1sCA (°)			
Pre	10.68 ± 3.37	10.63 ± 7.21	0.348
Post	9.89 ± 4.48	11.68 ± 6.83	0.213
F/U 2 years	11.30 ± 4.31	11.89 ± 6.36	0.661

*LG* low SCA group, *HG* high SCA group, *SCA* spino-cranial angle, *T1s* T1-Slope, *CA* C2–7 lordosis angle, *cSVA* C2–7 sagittal vertical axis, *T1sCA* T1s minus CA

**Table 6:** Comparison of cervical sagittal parameter changes

	LG (*n* = 41)	HG (*n* = 31)	*P* value
**SCA (°)**			
△CA (°) (pre vs post)	5.93 ± 6.77	2.30 ± 5.73	0.019
△CA (°) (pre vs F/U)	7.23 ± 6.40	3.02 ± 5.69	0.005
Pre vs F/U	< 0.001	0.006	
**T1s (°)**			
△T1s (°) (pre vs post)	− 3.21 ± 4.51	− 0.15 ± 4.80	0.007
△T1s (°) (pre vs F/U)	− 2.81 ± 3.69	− 0.69 ± 3.99	0.028
Pre vs F/U	< 0.001	0.383	
**CA (°)**			
△CA (°) (pre vs post)	− 2.43 ± 1.62	− 1.19 ± 2.46	0.005
△CA (°) (pre vs F/U)	− 3.43 ± 2.58	− 1.95 ± 2.93	0.017
Pre vs F/U	< 0.001	< 0.001	
**cSVA (mm)**			
△cSVA (mm) (Pre vs post)	5.57 ± 5.84	2.52 ± 6.89	0.055
△cSVA (mm) (Pre vs F/U)	7.51 ± 8.03	3.61 ± 5.76	0.019
Pre vs F/U	< 0.001	0.002	
**T1sCA (°)**			
△T1sCA (°) (pre vs post)	− 0.79 ± 4.73	1.05 ± 4.99	0.126
△T1sCA (°) (pre vs F/U)	0.62 ± 4.44	1.25 ± 4.82	0.453
Pre vs F/U	0.325	0.131	

*LG* low SCA group, *HG* high SCA group, *SCA* spino-cranial angle, *T1s* T1-slope, *CA* C2–7 lordosis angle, *cSVA* C2–7 sagittal vertical axis, *T1sCA* T1s minus CA

## Discussion

Multilevel cervical spondylotic myelopathy (MCSM) is a common disease, in which severe spinal cord compression is caused by the degeneration of multiple levels of intervertebral discs, joints, and ligaments and is often accompanied by severe clinical neurological deficits [8–11]. As a rather logical and popular surgical approach for the cure of MCSM, laminoplasty (LP) drifts the spinal cord backwards, resulting in indirect decompression [3, 12]. However, it may cause sagittal imbalance, such as the loss of cervical lordosis and the generation of kyphosis, which is associated with poor neck function [6, 13–15].

In recent years, spino-cranial angle (SCA), T1-slope (T1s), and C2–7 sagittal vertical axis (cSVA) have been considered the three most important parameters of the sagittal alignment for future study [4]. In our study, we followed the new sagittal parameter SCA to assess the association of SCA with other cervical sagittal parameters and postoperative recovery index. Under normal conditions, the sagittal angle of SCA fluctuates within a certain range (83° ± 9°) and has been reported to be affected by certain sagittal parameters, such as T1-slope (T1s) and C2-7 lordosis (CA) [5], consistent with our findings (T1s: *r* = − 0.795, *P* < 0.001; CA: *r* = − 0.857, *P* < 0.001). The T1s and CA values were significantly correlated with the NDI, according to previous literature [16]. In this study, we found that SCA was correlated with T1s (*r* = − 0.795, *P* < 0.001) and CA (*r* = − 0.857, *P* < 0.001) as well as directly correlated with the NDI scores in the high SCA group (pre: *r* = 0.876, *P* < 0.001; post: *r* = 0.414, *P* = 0.020, F/U: *r* = 0.431, *P* = 0.015), proving that SCA could significantly influence cervical alignment. Only when SCA exceeds a certain threshold does NDI increase as SCA increases. At the same time, the correlation of SCA with the NDI for patients with higher SCA increases with follow-up time (post vs F/U: 0.414 vs 0.431). Therefore, SCA can be considered another critical parameter for predicting imbalance because excessive SCA may result in significant cervical malalignment. Perhaps this is because the increase of SCA is accompanied by the decrease of C7 slope and the loss of lordosis after LP [17, 18], which would interfere with horizontal vision. Thus, patients may try to compensate cervical balance state by lowering T1s, resulting in the stretching of various muscles attached to the neck, which will trigger the threshold of pain and aggrandize energy consumption. Our study showed that, when SCA fluctuates within a certain range less than 85.2°, the cervical vertebra is considered to be within a state of compensation. However, when SCA exceeds the cut-off value, patients might be in a state of cervical decompensation, which results in poorer consequences. It also revealed a prominent positive correlation between SCA and the C2–7 sagittal vertical axis (cSVA) (*r* = 0.915, *P* < 0.001), and cSVA is known to be essential parameter for evaluating health-related outcomes [6, 19–23]. Excessive cSVA was more inclined to express a decrease in cervical lordosis [24–26] and a poorer recovery effect for multilevel cervical spondylotic myelopathy [18, 27]. The cSVA values of the high SCA group at all observed time points were significantly greater than those of the low SCA group (*P* < 0.001); thus, we considered that SCA increases with increased cSVA. An immoderate SCA may indicate mismatching of the cSVA, which may result in an imbalanced sagittal position of the spine and lead to poorer clinical outcomes. T1sCA (T1sCA = T1s − CA) is used to compensate for the changes of spinal alignment to maintain horizontal gaze (T1sCA ≤ 20°, balance; > 20°, imbalance) [28]. The value of T1sCA served as a sagittal balance criterion to classify SCA in this study. However, our study did not show a correlation between SCA and T1sCA (*r* = 0.072, *P* = 0.546). The difference between the two groups failed to reach significance (pre: *P* = 0.348; post: *P* = 0.213; F/U: *P* = 0.661), but T1sCA was always within a reasonable range (< 20°) before surgery, postoperatively, and at follow-up, which revealed that T1sCA could effectively compensate to maintain horizontal vision regardless of the SCA value. Meanwhile, patients in the low SCA group had a larger change in all cervical alignment parameters at the follow-up period, and patients in the higher SCA group lacked significant changes in radiographic and clinical indicators after LP surgery, which demonstrated that laminoplasty is an appropriate operating method for patients with a lower SCA and mainly ameliorates sagittal balance and relevant clinical outcomes for patients with lower SCA. The most likely reason could be that SCA, T1s, CA, and cSVA were within more suitable ranges in the low SCA group. Thus, we attached importance to the critical parameter SCA, which may be a good predictor of surgery planning. Our research is of great value in understanding the relationship between SCA and clinical outcomes after laminectomy. The value of SCA could be an appropriate indicator for assessing cervical sagittal balance and predicting the changes of cervical alignment after laminectomy.

Our study has certain limitations, such as its retrospective cohort study, shorter follow-up periods, and decreased patient data, which are needed to further confirm the aforementioned results.

## Conclusions

Patients with a lower spino-cranial angle (SCA) were more likely to show a larger sagittal change after laminoplasty and kept better sagittal balance, which led to a preferable surgical outcome. Laminoplasty could be an applicable choice for patients with lower SCA for multilevel cervical spondylotic myelopathy. SCA could be a good predictor of evaluating sagittal balance and planning surgery.

## Data Availability

The datasets generated and analyzed during the current study are available from the corresponding author on reasonable request.

## Abbreviations

MCSM: Multilevel cervical spondylotic myelopathy
SCA: Spino-cranial angle
T1s: T1-slope
CA: C2-C7 lordosis angle
cSVA: C2-C7 sagittal vertical axis
T1sCA: T1s minus CA
LP: Laminoplasty
JOA: Japanese Orthopaedic Association
NDI: Neck Disability Index
ROC: Receiver operating characteristics
HRQOL: Health-related quality of life
